# Grand challenges in colorectal and proctological surgery

**DOI:** 10.3389/fsurg.2023.1331877

**Published:** 2023-12-22

**Authors:** Gaetano Gallo, Marta Goglia

**Affiliations:** ^1^ Department of Surgery, Sapienza University of Rome, Rome, Italy; ^2^ Colorectal and Proctological Surgery Section, Frontiers in Surgery; ^3^ Department of Medical and Surgical Sciences and Translational Medicine, Faculty of Medicine and Psychology, Sapienza University of Rome, Rome, Italy

**Keywords:** colorectal surgery, proctology, pelvic floor, functional diseases, hemorrhoids, pilonidal disease, inflammatory bowel disease, rectal cancer

## Introduction

*“You must do the thing you think you cannot do”.* — *Eleanor Roosevelt*.

Colorectal surgery (CRS) and proctology represent a branch of general surgery that is becoming increasingly important and relevant. In recent decades, there have been so many innovations that have given coloproctological surgery an identity of its own. In recent years, innovations in major CRS have been revolutionizing healthcare and improving patient outcomes ranging from early diagnosis to the most modern treatment options.

One of the most significant breakthroughs in CRS has been the widespread adoption of minimally invasive techniques.

Laparoscopic surgery is now extremely widespread and practiced in CRS and the latter has completely revolutionized the field of major abdominal surgery by offering patients a less invasive approach that allows shorter hospital stays, reduced pain, and quicker recoveries. On the other hand, clinical research in the surgical field is moving in the direction of ever better instruments and technologies for the operating room. Recently, the single-incision laparoscopy technique has also been proposed in major colorectal surgery with total colectomy. This technique is often referred to as single-port laparoscopy or SILS (Single-Incision Laparoscopic Surgery) and represents a cutting-edge advancement in minimally invasive surgery (1). Another recently introduced new technology that allows an even less invasive approach to surgery with even fewer abdominal accesses is the Natural Orifice Transluminal Endoscopic Surgery (NOTES). Utilizing natural body openings such as the anus, vagina, and stomach for entry into the surgical area, it has been advocated as a method that leaves no visible scars. This technique allows access to various structures, including the distal rectum, which is challenging to approach using traditional laparoscopic methods. However, this approach has been associated with an increased risk of perioperative infection due to potential contamination and inadequate closure of access sites and the limited spatial orientation and the current skill level and instruments further compound the difficulty in learning and mastering this approach. Therefore, although several ideas have been proposed to secure the stapler anvil in NOTES, they have yet to gain widespread acceptance among surgeons (2).

What until recently was not feasible and suitable for major surgery, today, through surgical technological innovations is instead possible. Similarly, transanal total mesorectal excision (TaTME) offers the surgeon the possibility of combining a meticulous dissection of the mesorectal plane with a minimally invasive approach, ensuring a higher likelihood of complete tumour removal and reducing the risk of local recurrence (3). Transanal Transection and Single-Stapled anastomosis (TTSS) a new technique aims to gain and unify the best of the TME dissection and TaTME idea of transanal transection and single stapled anastomosis with promising good results in terms of efficacy and safety when compared with the traditional double stapled Knight Griffen technique and the TaTME anastomosis (4).

The Short-Stump and High-Anastomosis Pull-Through Procedure (SHiP) is an innovative approach recently introduced for treating rectal cancer. Unlike traditional methods, SHiP eliminates the need for creating a diverting ostomy, leading to a significant enhancement in patients' post-operative quality of life. Nonetheless, there is a possibility of functional post-operative changes, such as the occurrence of Low Anterior Rectal Resection Syndrome (LARS), anstomotic leak for ischemia of the rectal stump, anatostotic stenosis, and patient's discomfort from 7 to 14 days between the two operations. According to Bianco et al., with some technical modification, they made possible to avoid some of the drawbacks of the procedure. However, it needs further investigations (5, 6).

Robotic platforms for major abdominal surgery have recently been introduced into surgical technology. Their use to date is on an upward trend due to the continuous improvement in precision, manual dexterity and ergonomics, as well as the possibility of integrating the machine with other technologies. Despite this, some trials have shown that the robotic platform is not superior to the use of the laparoscopic technique in rectal surgery. However, robotic platforms are probably the innovation and technology of choice for the future (7).

Indeed, robotic platforms currently offer sensational innovations for colorectal surgery, ranging from enhanced maneuverability and precision, optimized machine ergonomics and the possibility of integrating artificial intelligence (AI) software, augmented reality and real-time three-dimensional reconstruction of the anatomy and pathology to be treated intraoperatively. Moreover, new robotic platforms are making their debut and introduce the flexible robotic surgery system enabling endoluminal and single incision surgery (EndoQuest Robotics) (8). Endoscopic surgery is in fact a new discipline that is increasingly gaining ground in the surgical field. Today it is possible to treat small colorectal lesions through new endoscopic devices and technologies with minimal invasiveness.

In addition, the integration of AI into the field of colorectal surgery represents a significant leap forward in the pursuit of precision medicine (9). Indeed, AI can be applied for multiple purposes for example for Diagnosis and Risk Prediction with the analysis of medical images, such as CT scans, MRIs, and endoscopy videos, to identify colorectal diseases, including polyps, tumors, and inflammatory conditions; as well as it can be employed for treatment planning by analyzing patient data AI can help tailor treatment plans to individual patients, optimizing surgical approaches and outcomes. AI can offer intraoperative assistance providing real-time guidance to the surgeon, representing a revolutionary tool for the experienced surgeon but also for the education of the surgeon in the first phase of the learning curve and the trainee. The design of ever finer and more effective AI software that can be applied to CRS is an exponentially growing field of study that will change modern surgery in the future, and its applications are manifold, ranging from pre-operative to early discharge patient management at home as per Enhanced Recovery After Surgery (ERAS) protocol. In the wake of the great success demonstrated by the ERAS protocol in patients selected for elective surgery, some authors have recently proposed the possibility of introducing major abdominal surgery in the outpatient setting. In fact, the practice of colectomy surgery in an outpatient setting has been proposed. Clearly, indications, protocols and limitations are currently under study (10, 11).

Furthermore, concerning oncological surgery news, the introduction of Complete Mesocolic Excision (CME) in right colon surgery represents a significant advancement. CME involves meticulous dissection and removal of the entire mesocolon, aiming for complete excision of the tumor along with its surrounding lymphatic drainage. This approach enhances oncological outcomes and reduces the risk of local recurrence. Implementing CME in right colon surgery reflects a commitment to precision and thoroughness, contributing to improved patient outcomes and long-term success in the surgical management of right-sided colon tumors. Among laparoscopic and robotic approach nonsignificant difference have been reported in Literature in terms of efficacy and safety. The advantage of a robotic approach may lie in the reduced learning curve and an increased penetration of minimally invasive approach to CME (12). Moreover, performing a targeted lateral lymph node (LLN) dissection alongside total mesorectal excision following neoadjuvant chemoradiotherapy (nCRT) is identified as a strategy to decrease lateral compartment recurrence and enhance overall survival in individuals diagnosed with rectal cancer featuring metastases in the LLN. Due to the promising results already published, this field deserves further efforts in investigating technical modification that could benefit the outcomes (13).

Total neoadjuvant therapy is a promising treatment for locally advanced rectal cancer, utilizing either short-course radiotherapy or long-course chemoradiotherapy. A recent paper comparing the two approaches demonstrated that long-course chemoradiotherapy with consolidation chemotherapy (HR 0.44, 95 per cent c.i. 0.20 to 0.99) exhibited higher disease-free survival than long-course chemoradiotherapy alone and that the pathological complete response rate and survival outcomes are similar for total neoadjuvant therapy with short-course radiotherapy or long-course chemoradiotherapy (14).

In the conservative management of early-stage rectal cancer, a key approach involves carefully considering non-surgical interventions or local excisional surgery instead of radical resections. Following neoadjuvant therapy, strategies for partial and complete response include close monitoring and potential adaptation of the treatment plan based on the individual patient's response. This may involve further surveillance, additional therapeutic modalities, or, in cases of complete response, the exploration of organ-preserving approaches to avoid unnecessary invasive surgery while ensuring effective cancer control. Local resection after neoadjuvant chemotherapy or chemoradiotherapy may be an effective alternative to radical surgery in patients with early and middle rectal cancer however, the tumors respond variably to this neoadjuvant therapy, and the mechanisms for response are still not completely understood (15–17).

In recent years, the possibility of using a pre-operative 3D printer has been introduced for the study of lesions that are candidates for surgery, but also for simulation and training of surgeons. Indeed, beneficial effects in terms of performance and understanding of surgery for students have been published. Currently, its evolution has been proposed with programs for three-dimensional reconstruction from medical images. Such 3D reconstruction represents a preoperative, but above all intraoperative tool that is possible and extremely useful in robotic surgery through augmented reality. These new technologies are equally if not more applicable to proctological surgery (18). For instance, 3D reconstructions could be an extraordinary tool for the visualization, study, and preoperative planning of complex anal fistulas. With regard to the latter, there is an ongoing debate on the use of minimally invasive techniques for their diagnosis and treatment (e.g., Video-Assisted-Anal-Fistula treatment, VAAFT) (19). Furthermore, minimally invasive surgery for pilonidal disease has gained wide popularity going from video-assisted ablation of pilonidal sinus (VAAPS) to endoscopic pilonidal sinus treatment (EPSiT), and laser assisted technique (20). Among proctological surgery, due to its frequency and impact on the quality of life, treatment of hemorrhoids has always played a central and crucial role. Enormous technological advancements have been recently introduced like the use of minimally invasive approaches as sclerotherapy for low grade disease, or doppler-guided hemorrhoidal dearterialization and laser aided techniques (21–23).

Increasing relevance has been given to the assessment of proctological benign pathologies in order to establish ways of objectively classify and tailor treatment on the basis of severity of symptoms and presentation of the disease. Indeed, scores for the classification of organic pathologies like updated Goligher scale for hemorrhoids or REALISE score for anal fissures as well as scores for functional pathologies as obstructed defecation syndrome score have been produced (24–26).

Obstructed defecation syndrome (ODS) is an extremely frequent condition, especially in the female population, whose treatment is not unambiguous and is often the subject of debate. In recent decades, a surgical procedure, the ventral retroplex, has been proposed as a treatment for ODS associated with external or tricompartmental haemorrhoidal prolapse. Recently, promising results have been reported in performing the procedure using a robotic platform (27). A lesson learnt from the COVID19 pandemic is that of the value and promising potential of telemedicine. Indeed, during the pandemic period, both for patients isolated because of the infection, and when all non-oncological and life-threatening urgent visits were postponed, telemedicine represented a bulwark of communication between patient and health personnel that denotes an area that must be supported. Moreover, the evolution of telemedicine in telesurgery have successfully addressed the challenge of geographical distance that once separated patients from surgeons capable of performing specific procedures. Telemedicine has already shown satisfactory results in the field of home monitoring and management of proctological patients and certainly, telemedicine can facilitate the patient's workflow in the diagnostic-therapeutic pathway and the empathic and caring doctor-patient relationship. Progress in robotic surgery, fiber optics, and computer technology holds the potential to overcome the limitations imposed by physical location, thus expanding patient access to the highest level of medical care. This evolution could effectively lead to the centralization of complex surgical procedures, ultimately enhancing patient safety (18, 28–30).

Some functional benign anal diseases, such as fecal incontinence, rectal prolapse, and hemorrhoidal disease are extremely common in the adult population and have a huge impact on the patient's quality of life. Over the last few years, great technological innovations and clinical research have led to enormous improvements in both clinical and strictly surgical treatment. However, it is still a young field that is open to innovation.

Colorectal and proctological surgery encompasses a broad spectrum of oncological pathologies with complex surgical treatment, but also extremely frequent benign functional pathologies with an enormous impact on the patient's quality of life. Both areas are now the subject of great interest due to their weight and frequency in the general population and have attracted the introduction of numerous new technologies and innovative proposals. The aim of this section is to bring together all the most innovative current research on the numerous areas of colorectal surgery.
